# Transcriptome and Targeted Hormone Metabolome Reveal the Mechanism of Flower Abscission in Soybeans Under Shade

**DOI:** 10.3390/ijms262110303

**Published:** 2025-10-23

**Authors:** Zhuorui Tan, Wenhui Han, Wanmin Mao, Xiang Wang, Shijun Li, Xinyang Luan, Xingdong Yao, Kai Guo, Futi Xie

**Affiliations:** 1 Soybean Research Institute, Shenyang Agricultural University, Shenyang 110866, China; 15804105950@163.com (Z.T.); 15593877379@163.com (W.H.); 18624389893@163.com (W.M.); wangx9403@163.com (X.W.); 19282402507@163.com (S.L.); ykarus@163.com (X.L.); 2 Key Laboratory of Soybean Biology of Chinese Education Ministry, Soybean Research Institute, Northeast Agricultural University, Harbin 150030, China; 3 College of Life Sciences, Shandong Normal University, Jinan 250014, China; guokaicc@163.com

**Keywords:** soybean, flower abscission, phytohormones, transcriptome

## Abstract

Shade-induced flower abscission in soybean plants is a significant factor limiting yield improvement. Under shaded conditions, significant differences exist in the flower abscission rates among different soybean varieties, but the regulatory mechanisms remain unclear. This study selected Tiedou 44 (T44) and Liaodou 32 (L32) as experimental materials. Results indicate that under shaded conditions, the flower abscission rate of T44 was significantly higher than that of L32. Physiological analysis revealed that cell wall degradation enzyme activity in T44 pedicels was significantly higher than in L32. Furthermore, compared to L32, T44 flowers under shade conditions exhibited significantly higher levels of IAA, IAA–amino acid conjugates, and ABA. The expression levels of *PIN* family genes (*GMPIN3C*, *GMPIN3D*, *PIN3A*, *GMPIN1A*, *GMPIN1B*, *GMPIN1C*, *GMPIN1D*, and *GMPIN1E*) in T44 were downregulated. These results suggest that the obstruction of auxin polar transport leads to auxin accumulation in flowers. This accumulation, in turn, triggers flower abscission. Additionally, *GH3* gene expression was upregulated in T44 compared to L32. GH3 proteins catalyze the conjugation of free auxin (IAA) with amino acids, forming inactive IAA–amino acid complexes. This significantly reduces the concentration of free IAA capable of inhibiting abscission in T44, making flowers more prone to abscission. This study provides crucial insights into the molecular regulatory mechanisms underlying flower abscission in soybean.

## 1. Introduction

Soybean (*Glycine max* (L.) Merr.) is a crucial grain, oil, and fodder crop that holds strategic importance for national economic development. With the continuous growth of the population and the sustained improvement in living standards, the demand for soybean is increasing. Therefore, boosting soybean production has become a global challenge. During the growth and development of soybean, frequent flower abscission occurs. This leads to a reduction in the number of effective pods and seeds per plant. This is a key factor limiting the production potential of soybean [1,2]. Especially when intercropped with corn, the shade cast by corn alters the light environment within the soybean canopy, increasing the rate of flower abscission and ultimately leading to reduced yields [3,4]. Shade-tolerant soybean varieties can achieve high yields in intercropping by reducing flower drop [5]. Under shaded conditions, significant differences exist in flower abscission rates among different soybean varieties. Compared to shade-intolerant soybean, shade-tolerant varieties exhibit lower flower abscission rates and achieve higher yields in intercropping systems. However, the regulatory mechanisms underlying these differences remain unclear.

Organ abscission is the process of organ separation, which plays a critical role in the life cycle of plants. Abscission has evolved as a successful strategy to adapt to the environment in response to developmental and environmental cues [6]. The process of abscission can affect vegetative and reproductive organs. It occurs by dissolving the cell wall at predetermined sites known as the abscission zone (AZ). The formation of the AZ is often related to stress or senescence [7]. The activities of cell wall dissolution enzymes such as polygalacturonases (PG), cellulases (CL), pectinmethylesterases (PME) and β-galactosidase (GAL) are related to the formation of the AZ [8,9]. The formation of the AZ is governed by plant hormones such as auxin (IAA), gibberellin (GA), abscisic acid (ABA) and ethylene (ET) [10].

Plant hormones play an important role in organ development and abscission [11,12]. In litchi, the genes involved in the biosynthesis, transport, metabolism, and signaling of ethylene, auxin, ABA, and GA are differentially regulated during organ abscission [13]. These findings further confirm that hormones are of particular importance to organ abscission as they act as effector molecules [14]. In general, ET and ABA act as abscission-accelerating signals [6,15], while IAA, GA and cytokinin (CTK) are thought to be abscission inhibitors [6,16]. Specifically, auxin regulates plant organ abscission through concentration gradients [17]. When auxin concentrations are elevated, ATP activity increases, leading to loosening of cell wall structures and ultimately causing organ abscission [18]. Additionally, studies on citrus fruits have revealed that ethylene accumulates extensively in the abscission zone. This promotes fruit drop [19]. Different plant varieties exhibit varying hormone levels. Cai et al. [9] demonstrated significant differences in ABA accumulation between distinct camellia strains (CF: abscission strain, CHF: nonabscission strain). This disparity results in differing degrees of leaf abscission. The abscission of soybean flowers and pods is co-regulated by endogenous hormone signaling [14]. Li et al. [20] investigated the effects of shade on the shedding of young lychee fruits and showed that shade induced the expression of genes involved in hormone synthesis and signal transduction in young fruits, suggesting that hormonal changes are important regulators of organ shedding under shade.

Current research primarily focuses on the physiological regulation of organ abscission by hormones and enzymes. Studies on the molecular mechanisms of organ abscission have also concentrated on model plants, such as grain shedding in rice [21,22], pod dehiscence in Arabidopsis [23,24], and flower and fruit drop in tomato [25,26]. Research on other species remains scarce. Therefore, identifying organ shed-ding-related genes across different species, elucidating their functions, and constructing molecular regulatory networks will be crucial for revealing the mechanisms of organ shedding and improving crop varieties [17]. Shade could increase flower abscission of soybeans, which would result in great loss for soybeans productivity. Therefore, it is of great importance to understand the mechanism of flower abscission and to formulate reasonable agronomic measures to prevent abscission under shade. In this study, two soybean cultivars with different flower abscission rates were used, and transcriptome sequencing was performed to compare and evaluate the differentially expressed genes in the flowers of the two cultivars. The objective was to determine the differences between these two soybean cultivars with respect to flower development, as well as the molecular pathways and key genes that control soybean flower abscission under shade. The results are expected to provide a theoretical basis for improving shade tolerance and yield of soybeans under shade.

## 2. Results

### 2.1. Effect of Shade Treatment on Soybean Flower and Pod Abscission

**Figure 1:**
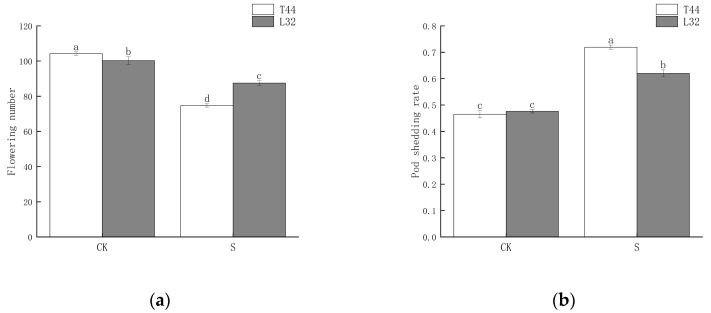
(**a**) Number of flowers on the soybean main stem; (**b**) soybean flower and pod abscission rate. Note: CK represents the natural light group; S represents the shade treatment group. The different lowercase letters (a, b, c, d) labeled above the bar charts indicate significant differences between groups (*p* < 0.05). The same letter indicates no significant difference between groups.

To investigate the effects of shade during the flowering period on soybean pod shedding, we selected two cultivars as research materials: the shade-intolerant variety T44 and the shade-tolerant variety L32. We statistically analyzed the number of flowers on the main stem of soybeans and the shedding rate of flowers and pods. The results showed that shade significantly reduced the number of flowers (Figure 1a). Between the two cultivars, the shade-intolerant T44 was significantly more affected than the shade-tolerant L32. Additionally, shade significantly increased the flower and pod abscission rate of soybeans (Figure 1b). Under natural light conditions (CK), there was no significant difference in the flower and pod abscission rates between the two cultivars; however, under shade conditions, the abscission rate of T44 was significantly higher than that of L32. Compared with the CK, the flower and pod abscission rates of T44 and L32 increased by 25.39% and 14.36%, respectively.

### 2.2. Effect of Shade Treatment on Soybean Abscission-Related Enzyme Activities

To investigate the effect of shade during the flowering stage on soybean flower abscission, the activities of β-galactosidase (β-GAL), polygalacturonase (PG), pectin methylesterase (PME), and cellulase (CL) were measured for each treatment.

**Figure 2:**
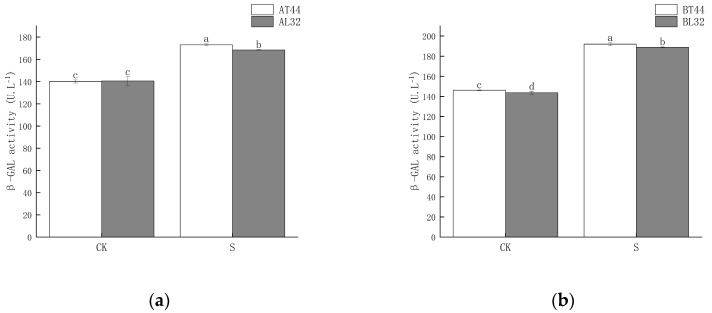
(**a**) β-GAL activity in pedicels at stage A; (**b**) β-GAL activity in pedicels at stage B. Note: CK represents the natural light group; S represents the shade treatment group; AT44, BT44, AL32, and BL32 denote T44 at stage A, T44 at stage B, L32 at stage A, and L32 at stage B, respectively. The different lowercase letters (a, b, c, d) labeled above the bar charts indicate significant differences between groups (*p* < 0.05). The same letter indicates no significant difference between groups.

Shade significantly increased β-GAL activity in soybean pedicels. Under shade treatment, β-GAL activity in the pedicels of L32 was significantly lower than that of T44 at both stages A and B. At stages A and B, compared with CK, β-GAL activity in L32 pedicels increased by 19.84% and 31.33%, respectively, while the increases in T44 were 23.53% and 31.28%, respectively. The results are shown in Figure 2.

**Figure 3:**
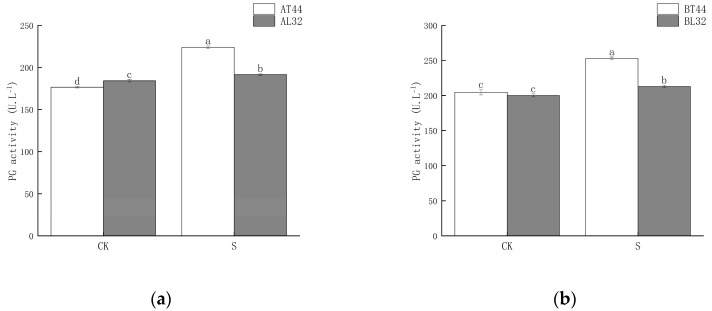
(**a**) PG activity in pedicels at stage A; (**b**) PG activity in pedicels at stage B. Note: The different lowercase letters (a, b, c, d) labeled above the bar charts indicate significant differences between groups (*p* < 0.05). The same letter indicates no significant difference between groups.

Shade significantly increased polygalacturonase (PG) activity in soybean pedicels. Under shade treatment, PG activity in L32 pedicels was significantly lower than that in T44 pedicels. At stages A and B, compared with CK, PG activity in L32 pedicels increased by 4.07% and 6.40%, respectively, whereas the increases in T44 pedicels were 26.91% and 23.56%, respectively. The results are shown in Figure 3.

**Figure 4:**
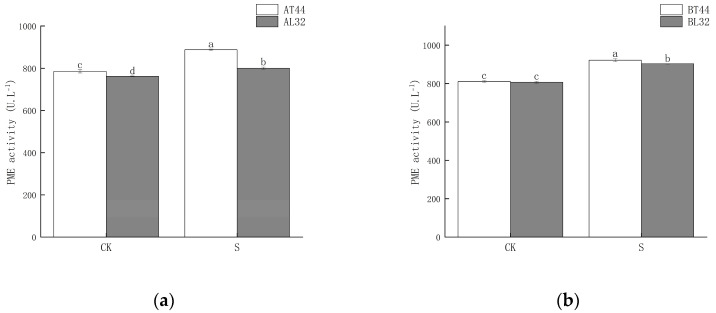
(**a**) PME activity in pedicels at stage A; (**b**) PME activity in pedicels at stage B. Note: The different lowercase letters (a, b, c, d) labeled above the bar charts indicate significant differences between groups (*p* < 0.05). The same letter indicates no significant difference between groups.

Shade significantly increased PME activity in soybean pedicels. Under shade treatment, PME activity in L32 pedicels was significantly lower than that in T44 pedicels. At stages A and B, compared with CK, PME activity in L32 pedicels increased by 5.04% and 11.86%, respectively, while the increases in T44 pedicels were 12.98% and 13.68%, respectively. The results are shown in Figure 4.

**Figure 5:**
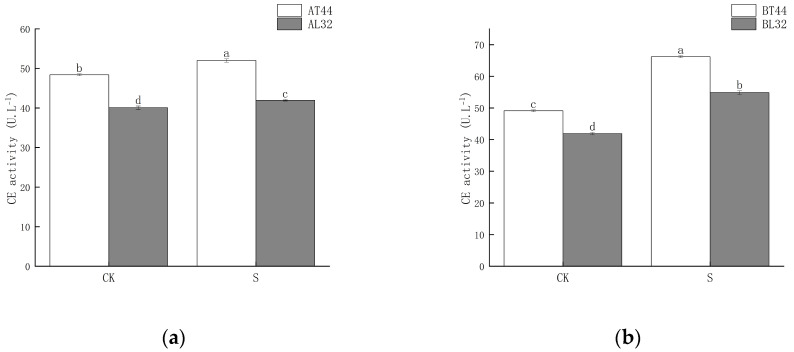
(**a**) CE activity in pedicels at stage A; (**b**) CE activity in pedicels at stage B. Note: The different lowercase letters (a, b, c, d) labeled above the bar charts indicate significant differences between groups (*p* < 0.05). The same letter indicates no significant difference between groups.

Shade significantly increased CE activity in soybean pedicels. Moreover, under both CK and shade treatments, CE activity in L32 pedicels was significantly lower than in T44 pedicels. At stages A and B, compared to CK, CE activity in L32 pedicels increased by 4.72% and 30.91%, respectively, while the increases in T44 pedicels were 7.49% and 34.81%, respectively. The results are shown in Figure 5.

### 2.3. Transcriptome Difference and Enrichment Analysis

To investigate the molecular mechanisms of shade-induced flower abscission, we conducted transcriptome analysis on cultivars T44 and L32. Samples were collected at two stages: stage A (full flowering) and stage B (Bloomed two days ago), under both control (CK) and shade treatments. The results showed that at stage A, comparing CK with shade treatment, 2381 expressed genes were identified in T44 and 4868 expressed genes in L32, with 966 genes co-expressed between the two cultivars (Figure S1a). At stage B, 3703 expressed genes were identified in T44 and 1276 expressed genes in L32 under CK versus shade treatment, with 450 co-expressed genes (Figure S1b). Using |log_2_(Fold Change)| ≥ 1 and adjusted *p*-value (Padj) ≤ 0.05 as criteria, 6554 differentially expressed genes (DEGs) were identified in the comparison of L32 versus T44 under shade treatment at stage A, including 2785 up-regulated genes and 3769 down-regulated genes (Figure S1c). At stage B, 13,132 DEGs were identified in the comparison of L32 vs. T44 under shade treatment, including 5383 up-regulated genes and 7749 down-regulated genes (Figure S1d).

GO functional analysis was performed on differentially expressed genes (DEGs), which were categorized into three groups: Molecular Function, Biological Process, and Cellular Component (Figure S2a,b). At stages A and B, the number of GO terms enriched by DEGs in Molecular Function was 1111 and 1204, respectively, among which 113 and 107 terms showed highly significant differences (*p* < 0.01). In Biological Process, 2811 and 3060 terms were enriched at stages A and B, respectively, including 204 and 304 extremely significantly different terms (*p* < 0.01). For Cellular Component, 435 and 509 terms were enriched, with 25 and 51 extremely significantly different terms (*p* < 0.01). KEGG pathway enrichment analysis revealed that the top three pathways with high enrichment and significance at both stages A and B were Metabolic pathways, Biosynthesis of secondary metabolites, and Plant hormone signal transduction (Figure S2c,d). Notably, the Plant hormone signal transduction pathway was enriched with 376 DEGs at stage A and 642 DEGs at stage B.

### 2.4. Changes in Auxin (IAA) Content and Expression of Its Response Genes

**Figure 6:**
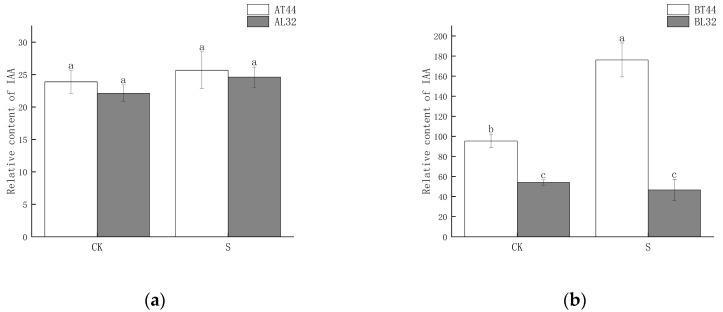

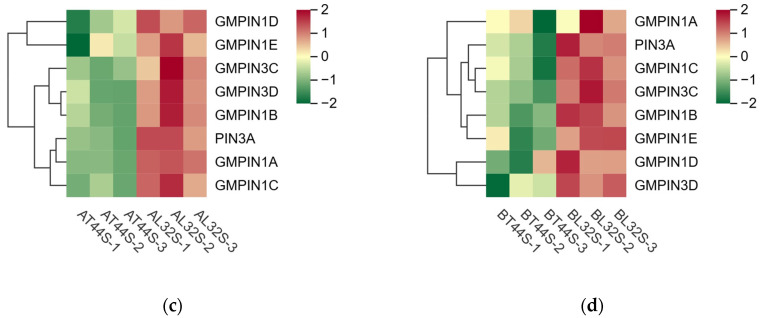
(**a**) Changes in IAA content in flowers under different treatments at stage A; (**b**) changes in IAA content in flowers under different treatments at stage B; (**c**) differential expression of PIN-related genes at stage A; (**d**) differential expression of PIN-related genes at stage B. Note: The different lowercase letters (a, b, c) labeled above the bar charts indicate significant differences between groups (*p* < 0.05). The same letter indicates no significant difference between groups.

Plant hormone detection was conducted on soybean flowers at stage A (full flowering) and stage B (two days after flowering). The results indicated that at stage A, there was no significant difference in IAA content among the different treatments (Figure 6a). However, at stage B, the IAA content differed significantly between T44 and L32 (Figure 6b). Under shade treatment, the IAA content of T44 was significantly higher than that of the CK, whereas no significant difference was observed in the IAA content of L32. This phenomenon may be related to the inhibition of IAA transport. To further investigate the cause of this phenomenon, a comparison was conducted on *PIN* family genes responsible for regulating the polar transport of IAA, identifying a total of eight *PIN* family genes (Figure 6c,d). Compared to L32, the expression of *PIN* family genes (*GMPIN3C*, *GMPIN3D*, *PIN3A*, *GMPIN1A*, *GMPIN1B*, *GMPIN1C*, *GMPIN1D*, and *GMPIN1E*) was significantly downregulated in T44. Furthermore, qRT-PCR validation was performed on randomly selected *PIN* family genes, and the results showed consistency between qRT-PCR and RNA sequencing data (Figure S3c–f).

**Figure 7:**
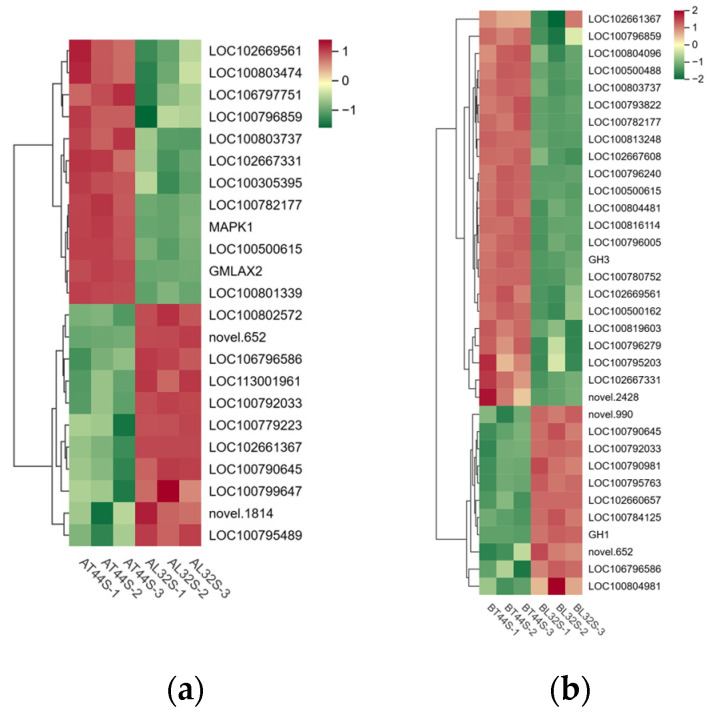
(**a**) Differential expression of IAA-related genes at stage A; (**b**) differential expression of IAA-related genes at stage B.

Meanwhile, the expression of genes involved in IAA signal transduction and other response factors was evaluated. At stage A, a total of 91 IAA-related genes were identified in the two cultivars under shade treatment, of which 11 genes were significantly upregulated and 13 genes were significantly downregulated in L32 (Figure 7a). At stage B, most genes involved in IAA signal transduction were downregulated in L32. A total of 149 IAA-related genes were detected in the two cultivars under shade treatment, with 6 genes significantly upregulated and 62 genes significantly downregulated in L32 (Figure 7b). From stage A to stage B, the expression levels of genes such as *LOC100792033*, *LOC106796586*, *novel.652*, *LOC102667331*, *LOC100500615*, *LOC100790645*, *LOC100803737*, *LOC102669561*, *LOC100782177*, and *LOC100796859* were significantly downregulated. At stage B, compared with stage A, the more significantly downregulated genes included *GH3*, *LOC102660657*, *LOC102667608*, *LOC100804481*, *LOC100813248*, *LOC100796279*, *LOC100819603*, *LOC100796005*, and *novel.2428.*

**Figure 8:**
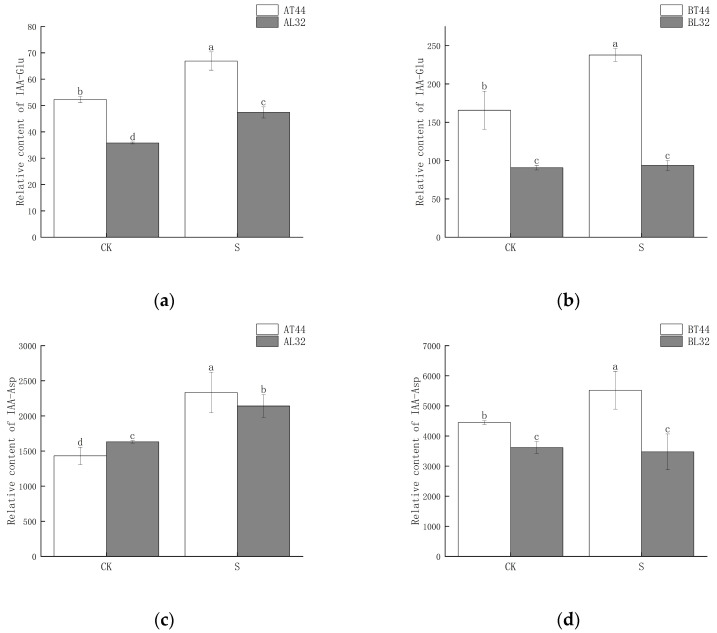

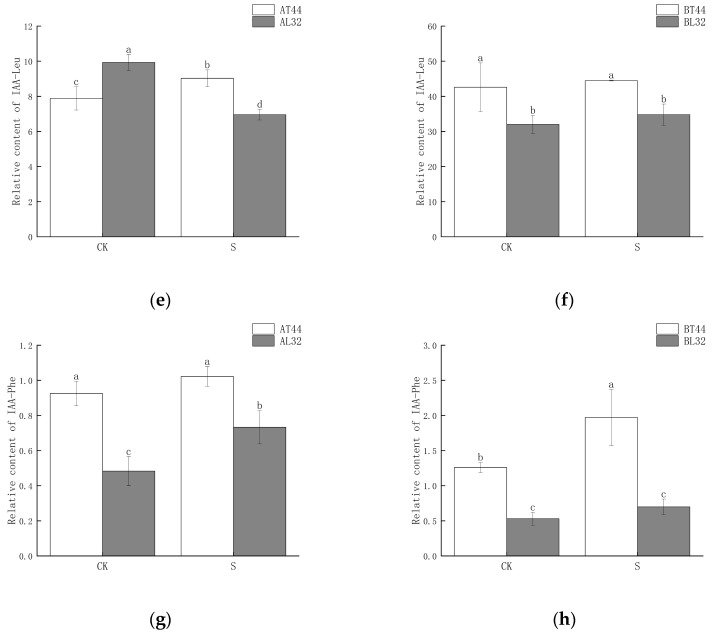
(**a**) Changes in IAA-Glu content in flowers under different treatments at stage A; (**b**) changes in IAA-Glu content in flowers under different treatments at stage B; (**c**) changes in IAA-Asp content in flowers under different treatments at stage A; (**d**) changes in IAA-Asp content in flowers under different treatments at stage B; (**e**) changes in IAA-Leu content in flowers under different treatments at stage A; (**f**) changes in IAA-Leu content in flowers under different treatments at stage B; (**g**) changes in IAA-Phe content in flowers under different treatments at stage A; (**h**) changes in IAA-Phe content in flowers under different treatments at stage B. Note: The different lowercase letters (a, b, c, d) labeled above the bar charts indicate significant differences between groups (*p* < 0.05). The same letter indicates no significant difference between groups.

Among these genes, *GH3* plays a crucial role in regulating IAA content in plants. Higher levels of *GH3* gene expression indicate the potential synthesis of greater amounts of GH3 protein. When IAA levels become excessively high, GH3 catalyzes the conjugation of IAA with amino acids to form inactive complexes. Furthermore, qRT-PCR validation results for the *GH3* gene were consistent with RNA sequencing findings (Figure S3g,h). Therefore, the content of IAA–amino acid conjugates was measured. The results showed that at both stages A and B, the IAA-Glu, IAA-Asp and IAA-Phe content in T44 was significantly higher than in L32 (Figure 8a–d,g,h). At stage B, under shade treatment, the IAA-Glu and IAA-Phe content in T44 was significantly higher than under CK, while no significant change was observed in L32. Moreover, the IAA-Asp content in T44 under shade treatment was significantly higher than under CK, whereas no significant change occurred in L32. Compared with CK, the IAA-Leu content in T44 under shade treatment was significantly increased at stage A, while it was significantly decreased in L32; at stage B, no significant differences in IAA-Leu content were observed among all treatments (Figure 8e,f). At stage B, the contents of IAA-Glu, IAA-Asp, IAA-Leu, and IAA-Phe in T44 were significantly higher than those in L32 under all treatments.

### 2.5. Hormone-Related Genes Involved in Soybean Flower Abscission

**Figure 9:**
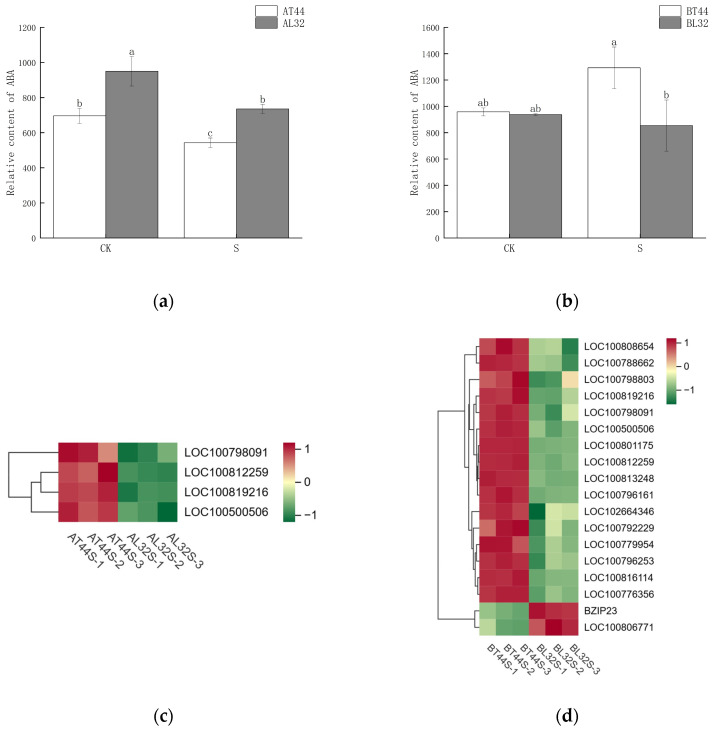
(**a**) Changes in ABA content in flowers under different treatments at stage A; (**b**) changes in ABA content in flowers under different treatments at stage B; (**c**) differential expression of ABA-related genes at stage A; (**d**) differential expression of ABA-related genes at stage B. Note: The different lowercase letters (a, b, c) labeled above the bar charts indicate significant differences between groups (*p* < 0.05). The same letter indicates no significant difference between groups.

Abscission is a complex physiological process that is regulated not by a single hormone but by the coordinated balance of multiple hormones. In addition to measuring IAA content, the content of abscisic acid (ABA) was also determined. The results are presented in Figure 9a,b. At stage A, the ABA content in T44 was significantly lower than in L32 under all treatments. At stage B, under CK, there was no significant difference in ABA content between T44 and L32; however, under shade treatment, the ABA content in T44 was significantly higher than in L32.

**Figure 10:**
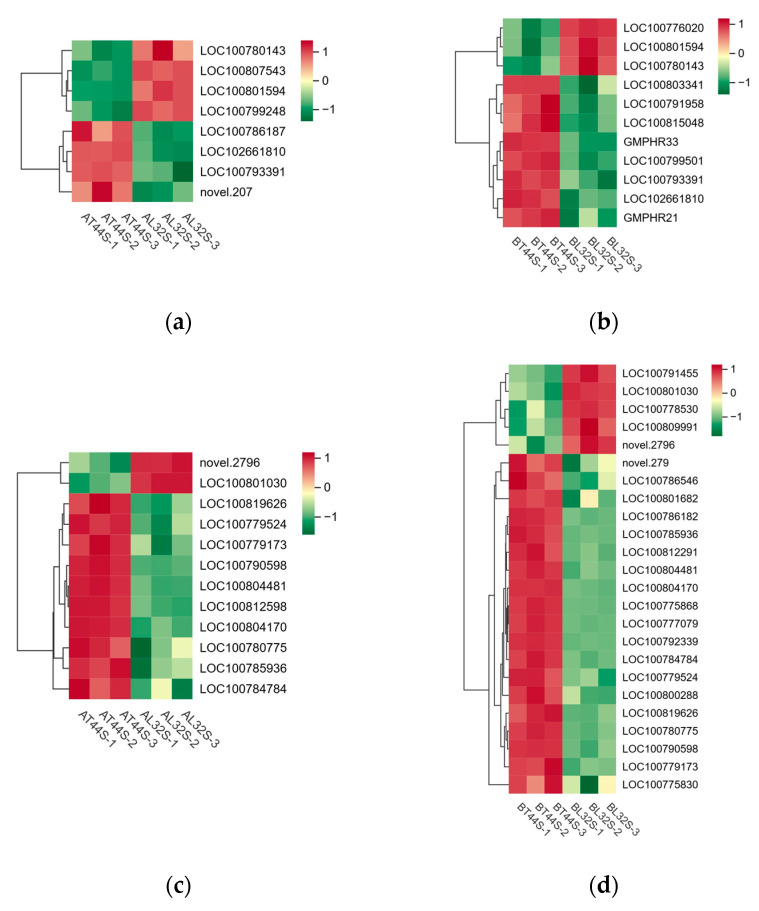

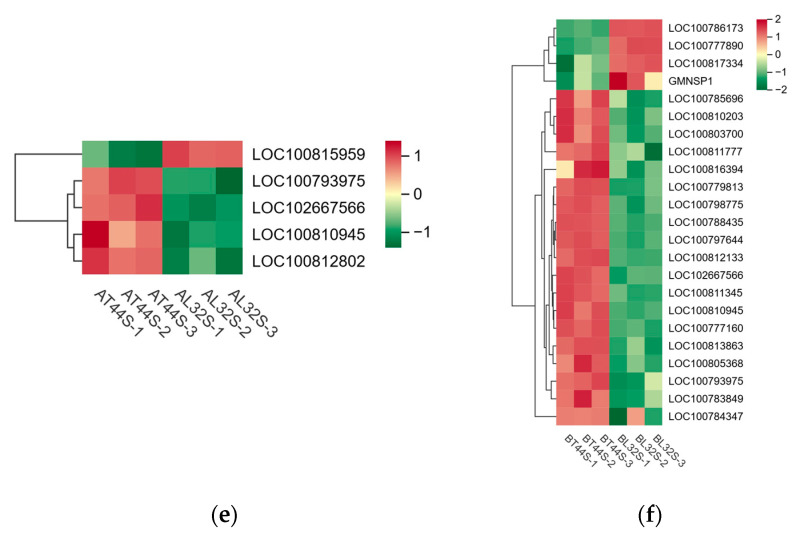
(**a**) Differential expression of CTK-related genes at stage A; (**b**) differential expression of CTK-related genes at stage B; (**c**) differential expression of ethylene-related genes at stage A; (**d**) differential expression of ethylene-related genes stage B; (**e**) differential expression of GA-related genes at stage A; (**f**) differential expression of GA-related genes at stage B.

Furthermore, transcriptome analysis revealed potential differences in the expression of key hormone-related genes involved in four plant endogenous signaling pathways: ABA (Figure 9c,d), cytokinins (CTKs), gibberellins (GAs), and ethylene (ET). Additionally, randomly selected ABA-related genes were validated via qRT-PCR, revealing consistent results between qRT-PCR and RNA sequencing data (Figure S3a,b). At stage A, there were 19 CTK-related genes, 34 GA-related genes, 13 ABA-responsive genes, and 29 ethylene-responsive genes (Figure 10a,b). At stage B, 45 CTK-related genes, 71 GA-related genes, 40 ABA-responsive genes, and 44 ethylene-responsive genes were identified (Figure 10c–f). Among ABA-related genes, the more significantly down-regulated genes at stage B compared with stage A included *LOC100796253*, *LOC100816114*, *LOC100779954*, *LOC100813248*, *LOC100808654*, *LOC100792229*, *LOC100776356*, *LOC100788662*, *LOC100796161*, *LOC100798803*, and *LOC100801175.* Among CTK-related genes, the more significantly down-regulated genes at stage B compared with stage A were *LOC100791958*, *GMPHR21*, *LOC100799501*, *GMPHR33*, *LOC100815048*, and *LOC100803341*. Most genes strongly expressed in the synthesis of GAs, ABA, and ethylene are positive regulators of flower abscission. The expression levels of most CTK-related genes, GA-related genes, ABA-responsive genes, and ethylene-responsive genes were significantly up-regulated in T44.

## 3. Discussion

During the reproductive growth stage, the reproductive organs of crops play a crucial role as the primary storage sites for the energy required for crop development, directly influencing crop yield. However, the abscission of plant reproductive organs (such as flower buds, flowers, and pods) is a widespread phenomenon [27]. Previous studies have demonstrated that environmental factors can affect both the number of flowers and the abscission rates of flowers and fruits in crops, ultimately impacting yield [28]. When soybeans experience shade stress during the growth and development stage, the total number of flowers is reduced [29], and the rates of flower and pod abscission are significantly increased [30], thereby reducing the number of effective pods per plant and affecting yield. In this study, the shade-tolerant cultivar L32 exhibited a significantly higher number of flowers and a significantly lower flower and pod abscission rate than the shade-intolerant cultivar T44 under shade conditions.

The abscission of plant organs is primarily triggered by the biosynthesis and secretion of hydrolases. The involvement of cell wall degradation-related enzymes is a key factor leading to the hydrolysis of the middle lamella and cell walls [31]. Previous studies have demonstrated a close relationship between abscission enzyme activity and the abscission process; the cell wall–degrading enzymes associated with abscission mainly include PG, CL, PME and β-GAL [32,33]. When plants are exposed to external environmental stress [6], the activity of abscission-related enzymes changes accordingly, thereby regulating the physiological and metabolic reactions related to abscission. In this study, shading significantly increased the activities of β-GAL, PG, PME, and CL. Under shaded conditions, the activities of all four enzymes in T44 pedicels were higher than those in L32. Furthermore, the magnitude of increase in β-GAL, PG, PME, and CL activities in T44 pedicels under shading was greater than that in L32. This indicates that T44 was more strongly affected by shading and exhibited greater susceptibility to abscission compared to L32.

Plant hormones are organic compounds produced in plants that regulate growth and developmental processes, such as flowering and fruiting, as well as responses to biotic and abiotic stresses [34]. Previous studies have confirmed that plant hormones play a crucial regulatory role in the abscission of plant organs and tissues [35]. The regulation of soybean flower and pod abscission by plant hormones is an extremely complex process, which is the result of the synergistic interaction of multiple hormones.

Auxin plays a key regulatory role in modulating plant organ abscission [36]. It primarily regulates abscission by altering the concentration gradient across the abscission zone [37]. When auxin polar transport is inhibited, the IAA gradient in the abscission zone (AZ) is disrupted, triggering activation of the distal abscission pathway and proximal ABA/ethylene synthesis, thereby promoting cell separation [18]. In bananas, IAA levels increase proximally (non-AZ) during abscission while decreasing distally (at the abscission zone), disrupting the auxin gradient and causing organ shedding [38]. In a tomato cold-induced flower abscission model, elevated IAA in the stamen and decreased IAA in the pistil (upstream of the abscission zone) concurrently lowered the abscission zone’s ethylene threshold, ultimately increasing the abscission rate [39]. RNA interference silencing of the *PIN1* gene in tomatoes increased auxin levels in the ovary while decreasing auxin levels in the abscission zone, thereby accelerating pedicel abscission. This indicates that auxin transport mediates the source-sink balance of auxin. This balance, in turn, is a critical factor influencing organ abscission [37]. This study found that during Stage B, under shading conditions, IAA significantly accumulated in T44 flowers. This led to a substantial decrease in the concentration of free IAA capable of inhibiting abscission zone formation in T44, thereby increasing the abscission zone’s sensitivity to ethylene and ultimately promoting flower abscission. Compared to L32, the *PIN* family genes responsible for auxin polar transport (*GMPIN3C*, *GMPIN3D*, *PIN3A*, *GMPIN1A*, *GMPIN1B*, *GMPIN1C*, *GMPIN1D*, and *GMPIN1E*) were all downregulated in T44 flowers, reducing IAA efflux. This disrupted the auxin concentration gradient across the abscission zone, destabilized the source-sink balance, and ultimately increased flower abscission rates.

**Figure 11:**
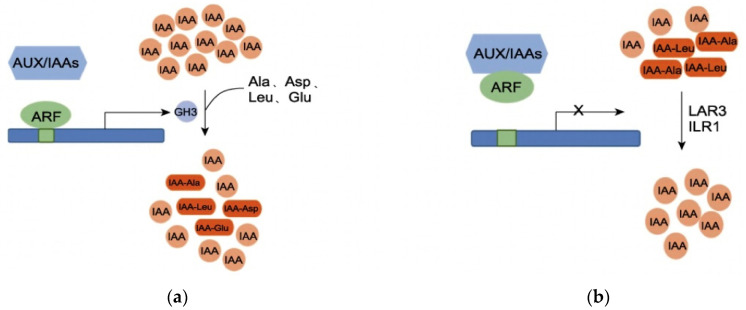
(**a**) Regulation of IAA dynamic balance at high IAA concentrations; (**b**) Regulation of IAA dynamic balance at low IAA concentrations. Note: *ARF* represents auxin response factor; Aux/IAA represents auxin/indole-3-acetic acid protein; GH3 represents Gretchen Hagen 3 protein; LAR3 and ILR1 represent amidohydrolases.

IAA activity is regulated by a dynamic balance, polar transport, and auxin response [40]. GH3 proteins play an important role in maintaining this dynamic balance of IAA in plants. When the IAA content in plants is high, GH3 proteins catalyze the conjugation of free IAA with amino acids (e.g., aspartic acid, glutamic acid) to form IAA–amino acid complexes, which inactivate IAA and reduce the concentration of free IAA [41]. When the IAA concentration in plants is low, IAA–amino acid complexes (acting as IAA storage pools) are hydrolyzed by proteases, and IAA is released again, re-entering the auxin signal transduction pathway to regulate the dynamic balance of auxin in plants [42]. At high IAA concentrations, auxin response factors (*ARFs*) dissociate from *Aux/IAA* dimers; *ARFs* then bind to corresponding auxin response elements (*AuxREs*) and activate *GH3* expression, thereby catalyzing the conjugation of IAA with amino acids [43,44]. When the concentration of indole-3-acetic acid (IAA) is low, *ARF* forms dimers with *Aux/IAAs* proteins, leading to the inactivation of *AuxREs*. Consequently, the transcription of the *GH3* gene is suppressed. Under these conditions, IAA-alanine (IAA-Ala) and IAA-leucine (IAA-Leu), which serve as auxin storage reservoirs, are hydrolyzed by amide hydrolases, resulting in the re-release of IAA (Figure 11a,b). At stage B, under shade treatment, the IAA content in T44 was relatively high compared to L32, and the expression of the *GH3* gene was upregulated. This catalyzed the conjugation of free IAA with glutamic acid, aspartic acid, leucine, and phenylalanine to form IAA-Glu, IAA-Asp, IAA-Leu, and IAA-Phe. Compared with CK, the content of these four IAA–amino acid complexes in T44 under shade treatment significantly increased and was significantly higher than that in L32. This indicates that free IAA in T44 is more extensively conjugated with amino acids. Among the formed complexes, IAA-Glu and IAA-Asp are degraded and inactivated via oxidative metabolic pathways, while IAA-Phe serves as an auxin storage pool. Consequently, the concentration of free IAA capable of inhibiting abscission is substantially reduced in T44. This reduction increases the sensitivity of the abscission zone to ethylene, thereby promoting flower abscission.

Abscisic acid (ABA) responds to environmental stresses and is involved in regulating the abscission of plant organs [45]. Previous studies have demonstrated that ABA accumulation promotes the abscission of plant organs. Carbohydrate starvation stress induces the up-regulated expression of ABA biosynthesis genes (*NCED* and *AAO*) in the pericarp and abscission zone, leading to ABA accumulation and subsequent fruit abscission [46,47,48]. In Arabidopsis thaliana, ABA has been shown to enhance the synthesis of polygalacturonase, which in turn increases the enzyme’s activity at an optimal pH, leading to the degradation of pectin in the cell wall [49]. This results in cell separation and ultimately leads to organ abscission. More specifically, ABA indirectly influences organ abscission by inhibiting auxin transport or stimulating ethylene production. Following ethephon treatment of young betel nut fruits, endogenous ABA content increases significantly, and the expression level of the auxin transport-related gene *PIN-LIKE7* is significantly down-regulated. This specifically inhibits the polar transport of auxin in the abscission layer of young fruits, reduces auxin content in the abscission zone, and ultimately triggers abscission [50]. In this study, under shading treatment during stage B, ABA content in T44 significantly increased. Compared to L32, numerous ABA-related genes were significantly upregulated in T44, while *PIN* family gene expression was downregulated. This disrupted the IAA gradient and stimulated ethylene production in response to ABA, ultimately increasing flower abscission rates.

Ethylene plays an important role as a regulator of cell separation during abscission and controls fruit abscission in coordination with various hormones. Studies on citrus [51,52], apples [46,53], and lychees [20,45] have demonstrated that ethylene and abscisic acid (ABA) are associated with fruit abscission caused by imbalances in carbohydrate metabolism. The accumulation of ABA in young fruits may be a response to sugar stress and is involved in the ethylene-activated abscission process [52]. Ethylene accumulates in the abscission zone and contributes to citrus fruit abscission [19]. Cytokinins are involved in regulating cell division and growth, and ethylene is believed to mediate the effects of cytokinins on abscission [54]. Beyond their primary roles in cell proliferation and differentiation, cytokinins also influence apical dominance, axillary bud formation, and leaf senescence. Maintaining a high concentration of cytokinins (>45 ng g^−1^) at the base of the flower stalk can significantly inhibit ethylene synthesis. Under shade treatment at stage B, the expression of a large number of CTK-related genes and ethylene-responsive genes was significantly upregulated in T44. These positive regulators may interact to accelerate cell division and separation in the abscission zone (AZ).

Gibberellins are involved in regulating cell expansion, fruit set, and growth [55]. Gibberellins (GAs) are tetracyclic diterpenoid plant hormones that may also play a secondary role in plant organ abscission. They are generally believed to influence organ abscission by modulating IAA synthesis; some studies further suggest that overexpression of gibberellin-related genes leads to organ abscission. In Camellia japonica, the up-regulated expression of gibberellin-related genes promotes GA accumulation, accelerates flower growth, and ultimately leads to early flower abscission [9]. Under shade treatment at stage B, the expression of a large number of GA-related genes was significantly upregulated in T44, which shortened the flower growth period and promoted early flower abscission. The above results provide a direction for elucidating the molecular regulatory mechanism of soybean flower abscission.

## 4. Materials and Methods

### 4.1. Plant Materials and Sample Collection

This study was conducted in 2024 at the South Field Experimental Station of Shenyang Agricultural University. The shade-tolerant variety L32 and the shade-intolerant variety T44 were used as experimental materials. T44 was developed in 1997 by the Tieling Soybean Research Institute of Liaoning Province through sexual hybridization, using Tie 93067-5 as the female parent and Tie 92022-8 as the male parent. L32 is a soybean variety bred by the Crop Research Institute of the Liaoning Academy of Agricultural Sciences, with parents Motto and Liao 21051. A split-plot design was employed, with the main plot being the shade treatment and the subplot being the variety. Shade treatment was applied to the soybeans starting from the flowering stage, with 50% artificial shade, and a natural light group (CK) was established. The experiment employed a pot-based method, with four seeds sown per pot and two plants retained per pot. Each treatment was replicated 30 times.

At the full flowering stage, two types of flowers were sampled: freshly opened flowers on the sampling day (designated as “A”) and flowers that had opened two days prior (designated as “B”). For each treatment, three biological replicates were collected. All sampled materials were immediately frozen in liquid nitrogen and then stored at −80 °C in an ultra-low temperature freezer. Additionally, starting from the initial flowering stage, three soybean plants with uniform growth were selected for each treatment. Data on flowers and pods on the main stem were recorded every other day until maturity. These data were used to determine the total number of flowers per plant, the number of pods per plant, and the rates of flower and pod abscission.

### 4.2. Determination of Abscission-Related Enzyme Activities

The activity of PG was determined using a detection kit (Solarbio Science & Technology Co., Ltd., Beijing, China). Samples stored at −80 °C were retrieved, and approximately 0.1 g of fresh sample was weighed. Pre-cooled extraction buffer (1 mL) was added, and the mixture was homogenized on ice. Subsequently, the samples were centrifuged at 16,000× *g* and 4 °C for 10 min. The supernatant was transferred to a new centrifuge tube and kept on ice for subsequent analysis. The 3,5-dinitrosalicylic acid (DNS) colorimetric method was used, and enzyme activity was calculated based on the absorbance measured at 540 nm.

CL activity was determined using a detection kit (Solarbio Science & Technology Co., Ltd., Beijing, China). Samples stored at −80 °C were retrieved, and approximately 0.1 g of fresh sample was weighed. One milliliter of pre-cooled extraction buffer (1 mL) was added, and the mixture was ground into a homogenate on ice. Centrifugation was then conducted at 8000× *g* and 4 °C for 10 min. The supernatant was transferred to a new centrifuge tube and kept on ice for determination. The DNS colorimetric method was used to measure the content of reducing sugars produced by the degradation of cellulose catalyzed by CL, and the enzyme activity was calculated based on the absorbance value at 540 nm.

The activity of β-GAL was measured using a detection kit (Solarbio Science & Technology Co., Ltd., Beijing, China). Samples stored at −80 °C were retrieved, and approximately 0.1 g of fresh sample was weighed. One milliliter of pre-cooled extraction buffer (1 mL) was added, and the mixture was ground into a homogenate on ice. The homogenate was centrifuged at 15,000× *g* and 4 °C for 10 min. The supernatant was transferred to a new centrifuge tube and kept on ice for analysis. β-GAL decomposes p-nitrophenyl-β-D-galactopyranoside to produce p-nitrophenol, and its activity was determined by measuring the rate of increase in absorbance at 400 nm.

The activity of pectin PME was determined using an enzyme-linked immunosorbent assay (ELISA) kit (Jiangsu Meibiao Biotechnology Co., Ltd., Wuxi City, China). Samples stored at −80 °C were retrieved, and approximately 0.1 g of fresh sample was weighed. The sample was ground into a powder using liquid nitrogen, and then 1 mL of pre-cooled phosphate-buffered saline (PBS, pH 7.4) was added and mixed thoroughly. The mixture was centrifuged at 2500 rpm and 4 °C for approximately 20 min. The supernatant was carefully collected, aliquoted into new centrifuge tubes, and kept on ice for analysis. The enzyme activity was determined using a double antibody sandwich method. Purified plant PME capture antibodies were coated onto microplates to prepare solid-phase antibodies. Plant PME was added to the coated microplates, followed by the addition of horseradish peroxidase (HRP)-labeled detection antibodies to form an antibody–antigen–enzyme-labeled antibody complex. After multiple washes with washing buffer, the substrate 3,3′,5,5′-tetramethylbenzidine (TMB) was added. HRP catalyzes the conversion of TMB into a blue product. This product then turns yellow under acidic conditions (pH < 7.0). The optical density (OD) was measured at 450 nm using a microplate reader (Tecan, Männedorf City, Switzerland), and PME activity in the samples was calculated using a standard curve.

### 4.3. Transcriptome Sequencing and Analysis

Samples used for transcriptome analysis included flowers at the full flowering stage: freshly opened flowers (A) and flowers that had opened two days earlier (B) from cultivars T44 and L32, subjected to CK and shade treatments. Each treatment had three biological replicates. Transcriptome sequencing was conducted by Metware Biotechnology Co., Ltd. (Wuhan City, China).

For gene expression quantification, the number of reads mapped to each gene was counted based on the alignment results and the positional information of genes on the reference genome. The number of fragments corresponding to a transcript depends on the amount of sequencing data (or mapped data), transcript length, and transcript expression level. To ensure that the fragment count accurately reflects the transcript expression level, normalization for both the number of mapped reads in the sample and the transcript length was necessary. Fragments Per Kilobase of transcript per Million mapped fragments (FPKM) was used as the metric to measure the expression level of transcripts or genes.

For differential gene screening, DESeq2 1.22.1 was used to perform differential expression analysis between sample groups with biological replicates, resulting in a set of differentially expressed genes (DEGs) between two biological conditions. Following the differential analysis, the Benjamini–Hochberg method was applied to adjust the *p*-values from the hypothesis tests to control the false discovery rate (FDR). The criteria for screening DEGs were |log_2_ fold change| ≥ 0.5 and FDR < 0.01.

For the functional annotation and enrichment analysis of differentially expressed genes (DEGs), the Kyoto Encyclopedia of Genes and Genomes (KEGG, https://www.genome.jp/kegg, accessed on 10 March 2025) serves as a comprehensive database integrating information on genomes, biological pathways, diseases, drugs, and chemical substances. KEGG seamlessly combines genomic data with high-level functional information, enabling systematic analysis of large datasets generated by genome sequencing and other high-throughput experimental technologies. After annotating genes using the KEGG database, the number of DEGs associated with each KEGG pathway was counted. Pathway enrichment analysis was conducted using the hypergeometric test, with KEGG pathways as units, to identify pathways significantly enriched in DEGs compared to the entire genomic background. Additionally, Gene Ontology (GO) is an international standard classification system for gene functions, established by the Gene Ontology Consortium. It is applicable to various species, de-fines and describes the functions of genes and proteins, and represents a dynamic vocabulary standard that is regularly updated with the advancement of research.

### 4.4. Analysis of Gene Expression by RT-qPCR

Gene-specific primers for qRT-PCR were designed using the NCBI online tool Primer-blast (https://blast.ncbi.nlm.nih.gov/Blast.cgi, accessed on 16 April 2025) (Table S1). Relative gene expression was quantified by quantitative real-time PCR (qRT-PCR) using SuperReal PreMix Plus (SYBR Green) from Takara (Dalian, China). The reactions were performed on an ABI PRISM 7500 Real-Time PCR System (Applied Biosystems, Thermo Fisher Scientific, Foster City, CA, USA).Cycling conditions were as follows: 95 °C for 30 s (1 cycle), followed by 95 °C for 5 s, 60 °C for 15 s, and 72 °C for 15 s (45 cycles). The soybean gene GmUKN1 (Glyma12g02310) was used as the internal reference gene in this study. Relative gene expression changes were analyzed using the 2^−ΔΔCt^ method [56,57]. All procedures were performed on three independent biological and technical repeats.

### 4.5. Data Processing and Analysis

All data were processed and analyzed using Microsoft Excel 2016. Statistical analysis was performed with SPSS 27.0, and Duncan’s multiple comparison test was used to determine the significance of differences (*p* < 0.05). Graphs were generated using Origin 2024b and Metware Cloud (https://cloud.metware.cn, accessed on 11 April 2025).

## 5. Conclusions

This study investigated the molecular mechanisms underlying flower abscission in shaded conditions using the shade-intolerant variety T44 and the shade-tolerant variety L32. Results revealed that shading significantly increased flower abscission rates in soybean, with T44 exhibiting a markedly higher rate than L32 under shaded conditions. Physiological analysis revealed that β-GAL, PG, PME, and CL activities in the pedicels of T44 were significantly higher than those in L32. Phytohormone analysis indicated that auxin content, IAA–amino acid complex content, and abscisic acid content in T44 flowers under shading were significantly higher than those in L32. Transcriptome analysis revealed that plant hormone signaling is one of the primary metabolic pathways in soybean response to shading stress. Compared to CK, T44 exhibited significantly elevated auxin content under shading treatment, whereas L32 showed no significant difference. Further investigation suggested this phenomenon may be associated with impaired IAA transport in T44. Compared to L32, *PIN* family genes (*GMPIN3C*, *GMPIN3D*, *PIN3A*, *GMPIN1A*, *GMPIN1B*, *GMPIN1C*, *GMPIN1D*, and *GMPIN1E*) were all downregulated in T44. These results indicate that disrupted auxin polar transport led to auxin accumulation in flowers, disrupting the auxin gradient across the abscission zone and subsequently causing flower abscission. Furthermore, compared to L32, *GH3* gene expression was upregulated in T44 flowers under shading. GH3 proteins catalyze the binding of free auxin to amino acids, forming inactive auxin–amino acid complexes (e.g., IAA-Glu and IAA-Asp). This significantly reduces the concentration of free auxin capable of inhibiting abscission in T44, promoting soybean flower abscission. Conversely, due to lower IAA concentrations in L32, stored IAA–amino acid complexes (e.g., IAA-Asp and IAA-Phe) were hydrolyzed by proteases, releasing additional free IAA to exert anti-abscission effects. Furthermore, under shading at stage B, T44 exhibited significantly higher ABA content than L32. The substantial upregulation of ABA-related genes in T44 likely stimulated ethylene production, which ultimately led to higher flower abscission rates. Transcriptomic analysis of T44 and L32 revealed abnormal activation of downstream response genes for hormones such as cytokinin, ethylene, and gibberellin in T44, indicating extensive signal transduction and transcriptional regulation in T44.

## Data Availability

This study is an original research paper. All data used in this study are included in the main text and Supplementary Materials of this paper.

## Supplementary Materials

The following supporting information can be downloaded at https://www.mdpi.com/article/10.3390/ijms262110303/s1.
