# Supplementary material for: Transcriptome and Targeted Hormone Metabolome Reveal the Mechanism of Flower Abscission in Soybeans Under Shade
Source: Int J Mol Sci. 2025 Oct 23;26(21):10303. doi: 10.3390/ijms262110303 (PMC12610579; doi:10.3390/ijms262110303)
Supplement: Supplementary file 1 [file ijms-26-10303-s001.zip › ijms-3935004-supplementary.pdf]

Table S1. The primer sequences for qRT-PCR.

Gene ID	Forward	Reverse
LOC100500506	CCATCACGCATGTTCAAGGC	TGAAGGGGCTAGCTTCATTG
PIN3A	TTCTATCCCGCAGTGCAGAC	CTGTTGGCAGGTTGAGCTGT
GMPIN1C	ATTACCATGCTGCTGGTGGA	AGGCCTCTTGGCATTAGCAT
GH3	CTCCGAGTTTCTCACCAGTTCT	GACACTGGACGTGCCACTAA

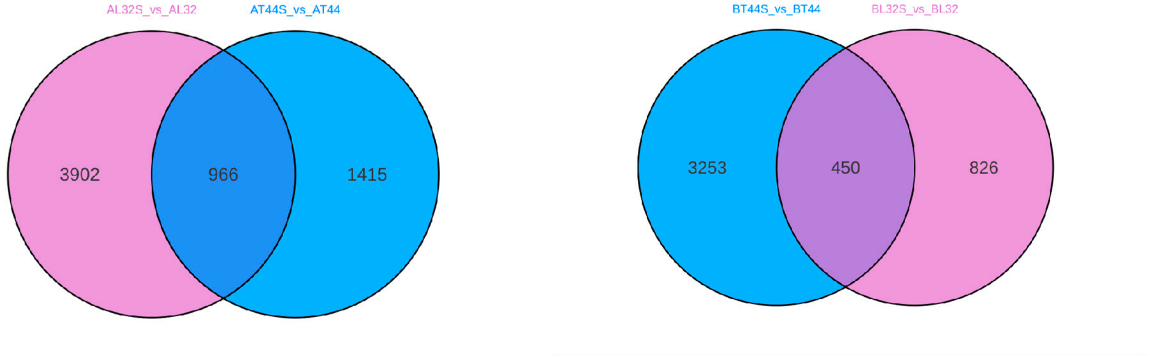

(**a**) (**b**)

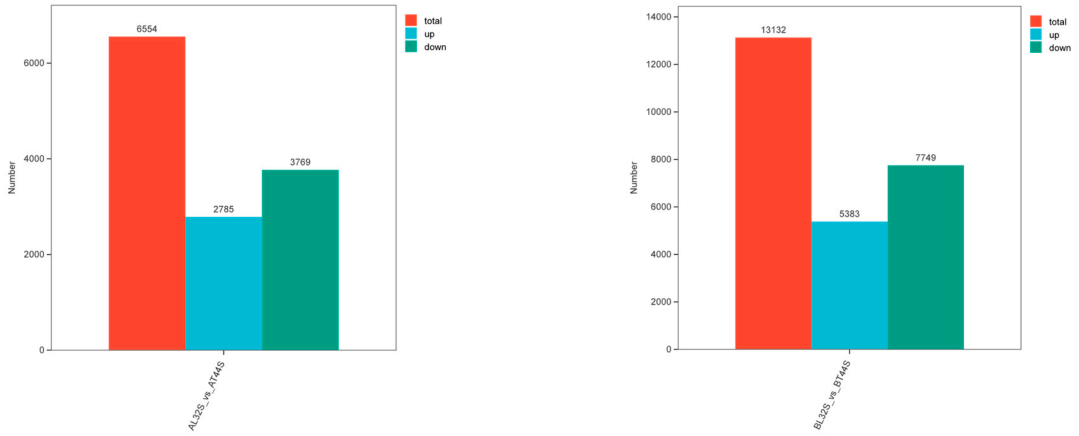

(**c**) (**d**)

Figure S1. (a) Venn diagram analysis of DEGs at stage A; (b) Venn diagram analysis of DEGs at stage B; (c) Number of up-regulated and down-regulated DEGs at stage A; (d) Number of up-regulated and down-regulated DEGs at stage B.

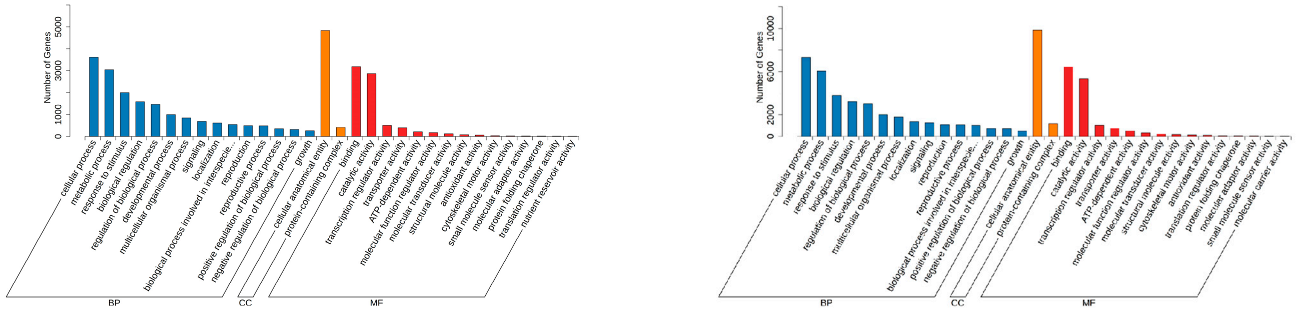

(**a**) (**b**)

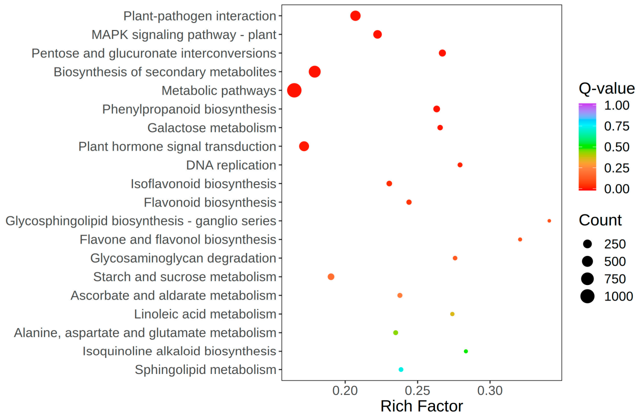

(**c**)

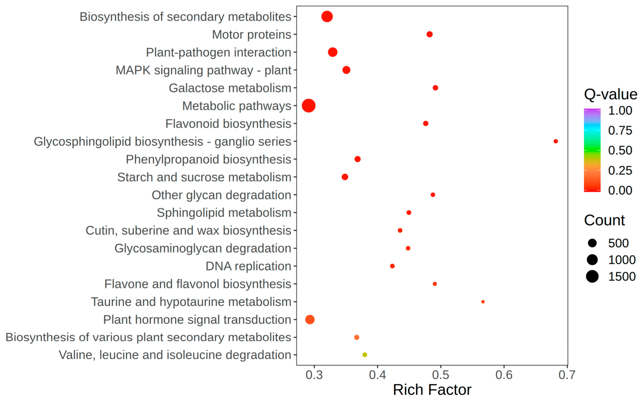

(**d**)

Figure S2. (a) GO functional analysis of DEGs at stage A; (b) GO functional analysis of DEGs at stage B; (c) KEGG pathway enrichment analysis of DEGs at stage A; (d) KEGG pathway enrichment analysis of DEGs at stage B.

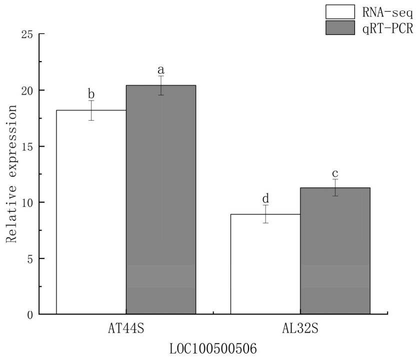

(**a**)

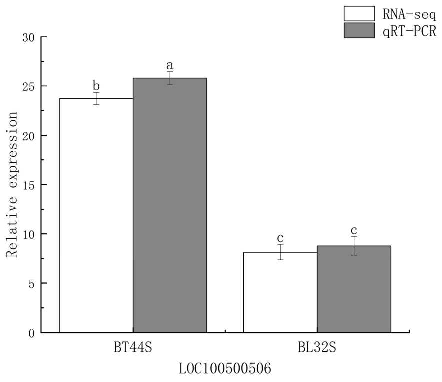

(**b**)

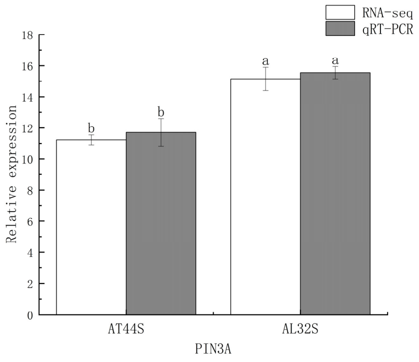

(**c**)

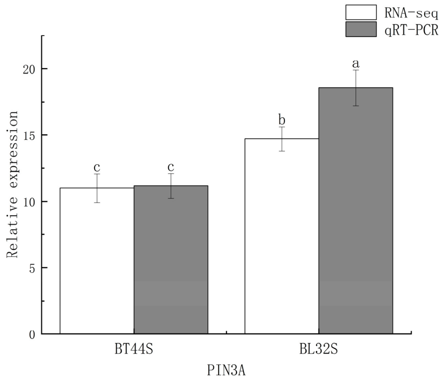

(**d**)

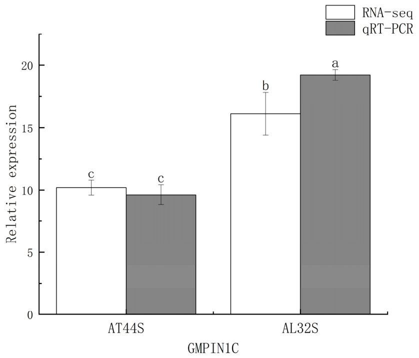

(**e**)

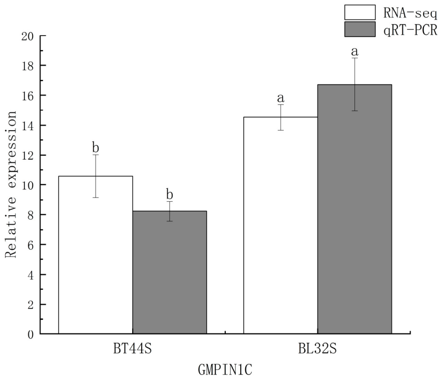

(**f**)

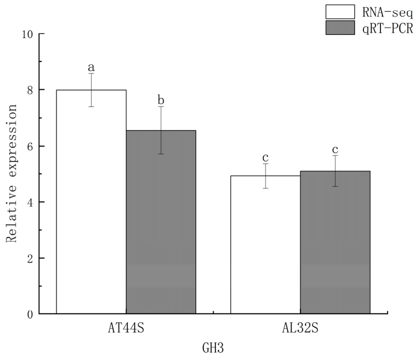

(**g**)

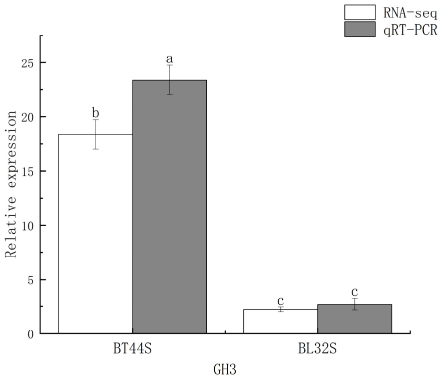

(**h**)

Figure S3. The qRT-PCR results confirm the transcriptome expression of the RNA sequencing.
